# Effects of Magnesium- and Cinnamon Essential Oil-Enriched Edible Gel Coatings on the Quality Parameters of Strawberries

**DOI:** 10.3390/foods15142534

**Published:** 2026-07-17

**Authors:** Gamze Alkaç, Enes Kavrut

**Affiliations:** 1 Innovative Food Technologies Development, Application, and Research Center, Iğdır University, 76000 Iğdır, Türkiye; alkacgamze0@gmail.com; 2 Food Engineering Department, Faculty of Engineering, Iğdır University, 76100 Iğdır, Türkiye; 3 Department of Gastronomy and Culinary Arts, Iğdır Faculty of Tourism, 76000 Iğdır, Türkiye

**Keywords:** edible coating, whey protein isolate, strawberry, essential oil, magnesium

## Abstract

This study aimed to determine the effects of whey protein isolate (WPI)-based edible gel coatings, enriched with different additives, on the quality parameters of strawberries (*Fragaria x ananassa*). The coating solutions were prepared in five different formulations: control (uncoated), WPI-based gel coating (GC), WPI + magnesium powder (GCMg), WPI + cinnamon essential oil (GCEo), and WPI + magnesium + cinnamon essential oil (GCMgEo). In the study, each experimental group was stored at 4 °C for 21 days and evaluated in terms of color parameters (L*, a*, b*, C*, h°, ∆E), weight loss, pH, Water soluble dry matter (WSDM), moisture content, redox potential (Eh), adhesion rate of the coating, decay percentage, texture analysis, and sensory properties. The results revealed that the GCMgEo group yielded the most successful outcomes in terms of color stability, oxidative resistance, and microbial control. However, sensory evaluation scores in this group were found to be lower compared to other groups. The highest overall acceptability scores were observed in the control group up to the 14th day. The coating applications were found to preserve the firmness and integrity of the strawberries, while the adhesion percentage increased with certain additives. Moreover, WPI-based coatings formed a protective film on the fruit surface, providing protection against compression and mechanical damage. These results indicate that while edible coatings slow down the ripening process, some additives may have negative effects on aroma and taste. As a result, WPI-based edible gel coatings have the potential to extend the shelf life of strawberries and reduce quality losses. The addition of magnesium and cinnamon essential oil enhances the functional performance of the coatings but requires sensory optimization. This study reveals that naturally derived coating systems can offer an eco-friendly and effective alternative for preserving fresh fruits.

## 1. Introduction

Strawberries (*Fragaria x ananassa*) are notable for their high nutritional value and flavor. However, they spoil quickly. The high water and sugar content, thin skin, and high respiration rate of strawberries make them extremely susceptible to physical, microbial, and chemical spoilage. This situation leads to significant quality losses and economic damage, particularly during the post-harvest period [1,2]. Ripe (fully mature) strawberries are soft and, in addition to high moisture levels, contain essential nutrients that can support the growth of microorganisms that cause spoilage. Disease outbreaks resulting from the consumption of contaminated strawberries are a cause for concern. Furthermore, their soft texture makes them highly susceptible to physical damage during storage or transportation [3,4]. Various post-harvest processing methods have been used to prevent microbial contamination of strawberries and extend their shelf life. Chemical treatment includes electrolysis, oxidizing water, organic acids, ozone, chlorinated compounds, ethanol vapor, and chlorination. Advanced techniques such as cold storage and modified atmosphere packaging (MAP) have been used, as well as traditional storage and packaging methods. More recently, biodegradable edible coatings have emerged as one of the most innovative applications in food preservation technology, offering both environmental sustainability and functional benefits [5,6,7,8]. WP-based films and coatings are versatile film-forming polymers that produce a flexible, colorless, odorless, and transparent film with a good oxygen barrier and heat-sealable properties. Compared to other film-forming polymers, WP possesses some distinctive characteristics (amphiphilic nature, 3D structure, denaturation, and electrostatic charges) [9]. It is also a potential carrier of active ingredients and functional additives (antioxidants, antimicrobials, nutrients, vitamins, and colorants) [10]. These components are used to improve the functional properties of the coating and extend the shelf life by maintaining the product’s quality characteristics [11].

Studies in the literature on post-harvest preservation of strawberries focus particularly on the development of edible coatings and the use of natural active ingredients. Vu et al. [12] comprehensively evaluated the potential of different edible coating materials to extend the shelf life of strawberries, while ref. [13] emphasized the importance of natural active ingredients in post-harvest preservation. Similarly, ref. [14] reported that whey protein isolate-based biodegradable coatings contributed to the preservation of strawberry quality. Sapper and Chiralt [15] showed that starch-based coatings formed a protective barrier on fruits and vegetables. In addition to their potential to act as barriers against oxygen, moisture and microorganisms, these coatings are enriched with natural antimicrobial and antioxidant components, thereby helping to preserve fruit quality [16,17]. These coatings, whose ingredients are recognized as Generally Recognized as Safe (GRAS) by the US Food and Drug Administration (FDA), add value to products by being enriched with functional ingredients [18,19]. In addition, plasticizers (sorbitol, glycerol) that are accepted under GRAS are added to the coatings [20].

Magnesium plays many important roles in cell physiology. For example, it serves as a cofactor for numerous enzymes. In the human body, it plays a critical role in the central nervous system and in biochemical reactions. It contributes to the digestive system by neutralizing stomach acid [21,22]. Furthermore, hydrogen (H_2_) gas is produced by reacting with water. The hydrogen is produced electrolytically using alkali or magnesium rods [23]. Hydrogen gas is highly valuable due to its small size, its ability to neutralize reactive oxygen species (ROS), and its capacity to act as a selective antioxidant [24]. In this regard, several foods, including green tea, beetroot, and grapes, have also been reported as sources or applications related to hydrogen-based functional effects [25]. When evaluating the eco-friendly properties of biodegradable films, both compliance with green chemistry principles and biodegradability are considered key parameters. Previous studies have evaluated these properties using the AGREE metric [26] and soil biodegradation tests [27]. Kavrut reported that whey protein isolate (WPI)-based films achieved an AGREE score of 0.82, indicating good compliance with green chemistry principles. In addition, Kavrut [28] and Kavrut and Çakmak [28,29] investigated the biodegradability of similar biodegradable films using a soil burial test and observed gradual structural degradation over a one-month period.

In this study, the effects of coatings prepared by adding functional additives such as magnesium powder and cinnamon essential oil to whey protein isolate (WPI)-based edible coating solutions on the physical, chemical, textural, and sensory properties of strawberries were investigated. Although there are many studies in the literature focusing specifically on chitosan-and starch-based coatings, the evaluation of WPI-based coatings incorporating minerals and essential oils remains limited [30,31]. Furthermore, most previous studies have focused on conventional coating formulations or a limited number of quality parameters. Therefore, the present study provides a more comprehensive approach by evaluating the effectiveness of a WPI-based edible coating enriched with magnesium powder and cinnamon essential oil through multiple quality attributes of strawberries. In this respect, the study aims to contribute to improving both shelf life and quality parameters through an innovative coating formulation. The findings of this study may provide a scientific basis for promoting the use of natural, eco-friendly, and functional coating materials in food packaging technology.

## 2. Materials and Methods

The strawberries (*Fragaria x ananassa*) used in this study were sourced from local producers in Bingöl Province, ensuring a uniform degree of ripeness. The strawberries were transported to the laboratory under cold chain conditions after harvest, and only those with intact physical integrity and uniform size and color were selected for analysis. The components used in the preparation of the coating solution, such as whey protein isolate (Hardline Nutrition Davisco Foods International Inc., Eden Prairie, MN, USA, 96% protein) and glycerol (Sigma-Aldrich, Waltham, MA, USA), were commercial products of high purity. Magnesium powder (Sigma-Aldrich, Darmstadt, Germany) and other chemicals, as well as all equipment used in the analyses, were sourced from the Iğdır University Centre for the Development, Application and Research of Innovative Food Technologies. Additionally, other chemicals (gallic acid, quercetin, Trolox, acetonitrile, methanol, ethanol, glacial acetic acid, orthophosphoric acid, and phenolic standard) were purchased from Sigma or Merck (Darmstadt, Germany).

### 2.1. Preparation of the Edible Coating Gel

The edible coating gel was prepared based on the method proposed by [17] with minor modifications. Whey protein isolate was used as the main component of the gel solution, and glycerol as the plasticizer. First, an aqueous solution of 5% (*w*/*v*) WPI (Hardline Nutrition Davisco Foods International Inc.) was prepared. 5 mL of glycerol (Sigma Aldrich) (1:1, volume) was added to the WPI solution and mixed with a magnetic stirrer. The resulting solution was then heated to 85 °C for 25 min and then cooled to room temperature. The initial °Brix value (10) of the solution was measured (Boeco Digital Abbe Refractometer, BOE 32400, Boeckel & Co., Hamburg, Germany). Subsequently, the solution was concentrated in an oven (35 °C) until it reached a gel-like consistency (18 °Brix). To enhance the coating solution’s adhesion to the strawberry surface and improve the coating process, the solution’s °Brix value was increased to achieve a gel-like consistency. Film solutions containing 12% WPI support the formation of a weak gel, whereas gels with a WPI content above 12% have a stronger structure [32].

### 2.2. Extraction of Cinnamon Essential Oil

The cinnamon was sourced from a local herbalist in Iğdır Province. It was ground into a powder using a grinder (Waring Commercial Blender 32BL80). To obtain the essential oil, an average of 50 g of cinnamon powder was weighed and placed into a 500 mL glass flask. Approximately 450 mL of distilled water was then added, and the mixture was placed in a Clevenger-type distillation device (Çalışkan, Ankara, Turkey). After a distillation process lasting approximately 1–3 h, the cinnamon essential oil was collected. Water was removed from the oil using anhydrous sodium sulphate (Na_2_SO_4_). The cinnamon essential oil was then transferred into dark-colored, screw-capped, light-proof bottles and stored in a refrigerator [17].

In terms of toxicity, cinnamon essential oil is a widely used ingredient in food applications as a natural antimicrobial and antioxidant. Indeed, the U.S. Food and Drug Administration (FDA) recognizes cinnamon essential oil as GRAS (Generally Recognized as Safe) [33].

### 2.3. Coating Strawberries with an Edible Whey Protein Isolate (Wpi)-Based Gel and the Formation of Experimental Groups

The prepared whey protein isolate (WPI)-based gel solutions were used for the coating process after achieving optimal viscosity and stability under aseptic conditions. All coating processes were carried out in a controlled laboratory environment. Strawberries with intact physical integrity, uniform size, and a similar degree of ripeness were selected for the experiments.

The strawberries were immersed in the respective gel solutions for 60 s, based on the optimum conditions determined in preliminary trials. After coating, excess gel was allowed to drain from the fruit surface, and the samples were placed in 15-compartment plastic egg trays with perforations created using a syringe tip. The coated strawberries were then air-dried in a sterile cabinet at room temperature. Both coated and uncoated (control) strawberries were stored under the same conditions in a refrigerator at 4 °C throughout the storage period. Five experimental groups were established: (i) an uncoated control group; (ii) a group coated with WPI-based gel only (GC); (iii) a group coated with WPI-based gel containing 160 mg/L magnesium powder (particle size < 0.1 mm; Sigma-Aldrich) (GCMg); (iv) a group coated with WPI-based gel containing cinnamon essential oil (GCEo); and (v) a group coated with WPI-based gel containing both magnesium powder and cinnamon essential oil (GCMgEo).

The magnesium-containing coating can release H_2_ gas as a result of the reaction between magnesium and the moisture in the strawberries, as shown in the following reaction:

$$Mg+2H_{2}O\to Mg({OH)}_{2}+H_{2}$$

The magnesium used in this study was magnesium powder (Mg, elemental form; Sigma-Aldrich) and was added to the coating solution (Section 2.1) at a concentration of 160 mg/L. Magnesium was added slowly and controllably to the pre-prepared WPI-based gel solution using a magnetic stirrer. Stirring was continued for approximately 5 min until a homogeneous distribution was achieved. The solvent used was distilled water, which served as the basic dispersion medium in the preparation of the entire coating system. The pH value was measured (pH: 6.92) and recorded during the preparation of the coating solution.

Cinnamon essential oil was emulsified before being directly added to the coating matrix. For the emulsification process, a pre-emulsion was prepared by mixing 10 mL of distilled water, 100 µL of Tween 80 (Sigma-Aldrich), and 0.5 mL of cinnamon essential oil at high speed in a vortex mixer (2 min, at room temperature).

The concentration of cinnamon essential oil was determined based on preliminary trials conducted in the range of 0.25–1.0% (*w*/*v*). In these preliminary trials, strawberries were immersed in solutions containing different concentrations of essential oil and then evaluated through preliminary sensory analysis. Sensory evaluation included taste, odor, and bitterness (perceived oral irritation) parameters. The results showed that the 0.5% (*w*/*v*) concentration provided acceptable sensory characteristics and offered the best balance among the tested concentrations. Therefore, the 0.5% (*w*/*v*) cinnamon essential oil concentration was selected for subsequent experimental studies. Each experimental group contained an equal number of samples, and all procedures and storage conditions were performed under the same environmental conditions to ensure the reliability and comparability of the results.

### 2.4. Analyses

#### 2.4.1. Color Analysis

The color characteristics of the strawberries were analyzed using the International Commission on Illumination (CIE) L*, a*, b*, C*, h° color space. Measurements were taken using a Konica Minolta CR-A501 (Tokyo, Japan) color measurement device; the device was calibrated with a standard white plate prior to each measurement. The values obtained were as follows [34]: L*: Lightness (0: black–100: white), a*: Green (−)/Red (+), b*: Blue (−)/Yellow (+), C*: Chroma, h°: hue angle. Total color changes (ΔE) were calculated as follows:

$$∆E=\sqrt{{\left(L_{0}^{*}-L_{t}^{*}\right)}^{2}+{\left(a_{0}^{*}-a_{t}^{*}\right)}^{2}+{\left(b_{0}^{*}-b_{t}^{*}\right)}^{2}}$$

where

L*_0_, a*_0_, b*_0_ represent the color values of the strawberries on the 0th day of storage.
L*_t_, a*_t_, b*_t_ represent the color values of the strawberries, except on the 0th day of storage.

For color analysis, strawberries of the same maturity stage and homogeneous external color were selected. Color measurements were taken on days 0, 7, 14, and 21 of storage. Before measurement, each strawberry was cut longitudinally into two halves, and the outer surface of each half was placed against the measurement aperture of the colorimeter. Color values were recorded. For each treatment group at each storage interval, six strawberries placed in egg trays were analyzed separately. Three consecutive readings were taken from the same point on the outer surface of each strawberry, and the mean L, a, b*, C*, and h° values were used for statistical analysis.

#### 2.4.2. pH Analysis

pH values were determined using a multiparameter measurement instrument (Consort C3040, Bruxelles, Belgium) with an SP10R pH electrode probe [35]. The instrument was calibrated using buffer solutions of pH 4.0 and 7.0 before analysis. A total of 10 g of homogenized strawberry sample was added to a beaker containing 100 mL of distilled water. The samples were homogenized for 1 min using an Ultraturrax (IKA^®^ T18 digital ULTRA-TURRAX^®^, IKA-Werke GmbH & Co., Staufen, Germany), and measurements were taken.

#### 2.4.3. Water Soluble Dry Matter (WSDM)

Measurements were performed using a digital refractometer (Boeco Digital Abbe Refractometer, BOE 32400, Germany). Before analysis, the instrument was calibrated with pure water. For the analyses, strawberry samples from each group were homogenized to obtain a homogeneous slurry. 1–2 drops of the resulting sample were placed on the instrument’s prism. The water-soluble dry matter content (%) was then recorded.

#### 2.4.4. Eh Analysis

Measurements were performed using a Consort C3040 (Belgium) multiparameter meter. The Eh electrode was cleaned with aluminum oxide (Al_2_O_3_) and calibrated prior to measurement [36].

#### 2.4.5. Moisture Analysis

Moisture content is a critical parameter affecting texture, microbial growth, and shelf life. Approximately 50 g of each strawberry sample were taken, and their moisture content (%) was determined using a rapid moisture analyzer (MOC63u, Shimadzu, Kyoto, Japan).

#### 2.4.6. Weight Loss Analysis

The weights of packaged and control samples were recorded weekly during storage using an electronic precision balance with a sensitivity of 0.001 g (A&D Company Ltd., FZ-500i, Tokyo, Japan). The weight loss (%) of the samples was calculated using the following formula, based on the initial weight of the product [37].

$$Weight\;Loss\;(\%)=\frac{\left(Initial\;Weight-Final\;Weight\right)}{Initial\;Weight}\times 100$$

#### 2.4.7. Coating Adhesion Percentage

The adhesion of the edible gel coating to the strawberry surface is a critical parameter in determining the coating’s efficacy and stability. The weights of coated and uncoated strawberries were measured, and the adhesion percentage was calculated using the following formula [38].

$$Adhesion\;Percentage\;(\%)=\frac{\left(K-I\right)}{I}\times 100$$

where:

K: Weight of the coated strawberry (g),
I: Weight of the uncoated strawberry (g).

#### 2.4.8. Percentage of Degradation

The proportion of fruits affected by fungal, mold, or other microbial growth was monitored throughout the storage period and expressed as a percentage. For each treatment group, observations were conducted using packs containing six fruits (2 replicates in 3 independent trials). The decay percentage was calculated by dividing the number of spoiled fruits by the initial total number of fruits and multiplying the result by 100 [39]. The percentage of spoilage of the strawberries was calculated using the following formula:

$$Decay\;Percentage\;(\%)=\frac{N_{decayed\;fruits}}{N_{initial\;total\;fruits}}\times 100$$

where:

*N_decayed fruits_*: number of fruits showing fungal or microbial decay
*N_initial total fruits_*: total number of fruits at the beginning of storage.

This equation was used to calculate the percentage of decayed fruits in both coated and uncoated samples.

#### 2.4.9. Extraction

One gram of freeze-dried strawberry sample was mixed with 20 mL of 75% (*v*/*v*) ethanol. The mixture was then placed in a shaking incubator (HZQ-X300, China). It was incubated for 24 h at 120 rpm and 35 °C. Three batches of each strawberry extract were prepared. The alcoholic (75% ethanol) filtrate was dried at 35 °C using a rotary evaporator (Heidolph Hei-VAP, Schwabach, Germany). The dried extracts were stored at −80 °C until further analysis [40].

#### 2.4.10. HPLC Analysis

The phenolic profile was determined using an HPLC instrument, with minor modifications to the method described by Engin et al. [40]. 20 μL of extract samples were injected into a C18 ACE Generix column (250 × 4.6 mm, 5 μm particle size) at a temperature of 25 °C. Two mobile phases were used: solvent A (0.1% orthophosphoric acid in water) and solvent B (acetonitrile). The initial mobile phase consisted of 83% A and 17% B and was used at a flow rate of 0.8 mL/min and an injection volume of 20 μL. The total run time was 40 min. The gradient program started with 83% A and then changed to 60% and 83% at 30 min and 35 min, respectively. Phenolic compounds were detected at 350/200 nm using a UV-DAD detector.

#### 2.4.11. Texture Profile Analysis (TPA)

Texture profile analyses (TPA) of strawberry samples were carried out using the TA.XT2 Tissue Analysis System (Stable Micro Systems, Godalming, UK), which features a 5 kg load cell. P/0.25S spherical probes were used for the analyses. The test parameters were set as follows: 1 mm/s initial velocity, 1 mm/s final velocity, 10 mm/s test velocity, 25% compression strain, and a 5 s interval between compressions. Two penetration tests were performed for each strawberry sample [41].

#### 2.4.12. Sensory Analysis

A descriptive sensory analysis method was used to determine the sensory characteristics of the strawberries [42]. In the evaluation test, which involved 10 trained panelists (academic staff and postgraduate students from the Department of Food Engineering at Iğdır University), the sensory characteristics of the strawberries throughout storage was scored in accordance with Table S1. This study was approved by the Scientific Research Publication Ethics Committee of Iğdır University (approval number: E-37077861-100-210762; Date: 7 March 2026).

#### 2.4.13. Statistical Analysis

Statistical evaluation of the data obtained from strawberry fruit was performed using the IBM SPSS 25 software package. Differences between groups were determined using one-way analysis of variance (ANOVA). Duncan’s multiple comparison test was used for pairwise comparisons of groups. Significance levels were expressed as *p* < 0.05. Principal Component Analysis (PCA) was performed using SIMCA 14.1 (MKS UMETRICS, Umea, Sweden) to evaluate the multivariate relationships among the texture parameters of the samples. Additionally, Origin 2026 software was used for the graphical representation of sensory classification. The study was conducted in triplicate.

## 3. Results and Discussion

### 3.1. Analyses

#### 3.1.1. Color Analysis

Color is one of the most important characteristics of fruit that directly influences consumers’ perception of quality; in strawberries in particular, a bright, uniform red color is a key marker of freshness and ripeness [43]. The color parameters (L*, a*, b*, C* and h°) varied depending on both the treatment and the storage period (Table 1). While the brightness (L*) of the control group decreased throughout storage, this decline became particularly pronounced in the GC group on day 21 (*p* < 0.05). In contrast, brightness remained more stable in the GCMg, GCEo, and GCMgEo groups (*p* > 0.05). This suggests that magnesium and essential oil can improve the coating structure without altering or degrading the natural surface color and brightness of the food [44,45]. This is due to the fact that magnesium and essential oils strengthen the coating matrix, delaying water loss and enzymatic browning (oxidation).

Redness (a*) values were generally preserved across all groups (*p* > 0.05). However, the coated groups exhibited better preservation of redness at the end of storage. Although no significant differences were observed in yellowness (b*) values, the lower values obtained in the coated groups at the end of storage indicate that the pigments were better preserved. Chroma (C*) remained unchanged in the control group but decreased in the GC group (*p* < 0.05). In contrast, it remained more stable in the GCMg, GCEo, and GCMgEo groups [46]. While the hue angle (h°) decreased in the GC group, it remained unchanged in the other coated groups (*p* > 0.05). These findings suggest that the H_2_ gas generated by magnesium may reduce color deterioration by slowing enzymatic activity and, when combined with essential oil, may further improve pigment stability [47]. Overall, multi-component coatings (Mg + essential oil) appeared to be more effective in maintaining color stability and limiting quality deterioration during storage [48,49].

During storage, strawberries undergo color change (ΔE) due to respiration, water loss, and changes in anthocyanins and other pigments [50]. Table 1 shows that ΔE values increase in all coated groups during storage (*p* > 0.05). However, at the end of storage, the highest color change in coated strawberries was observed in the GCMgEo group (12.99), and the lowest color change (8.96) was observed in the GC group. However, the lowest values were detected in the coated groups at the beginning of storage (day 7). As storage progressed, the color change in the groups also differed. These differences may be associated with the effects of magnesium and essential oil incorporation on the optical properties of the coating matrix and pigment stability during storage.

#### 3.1.2. pH Values of Strawberries

In strawberries, pH is a key quality indicator closely linked to ripeness, respiration, organic acid metabolism and microbial activity. Changes in pH during storage arise primarily from organic acid consumption and cellular degradation, and pH stability is critical for shelf life [51]. In the study, pH values in the control group fluctuated throughout storage (*p* < 0.05), indicating that acid metabolism proceeded irregularly (Table S2). In the GC group, however, the pH remained stable initially but rose to 3.64 on day 21, showing a significant increase (*p* < 0.05); this suggests that the protein-based coating was insufficient for long-term protection. In contrast, pH values in the GCMg and GCEo groups remained constant throughout storage (*p* > 0.05), which may be attributed to the buffering effect of magnesium and the suppression of microbial and metabolic activity by essential oils. In the GCMgEo group, however, the pH rose to 3.99 on day 21, differing from the other groups (*p* < 0.05); this increase indicates that the consumption of organic acids may accelerate during extended storage, potentially leading to changes in the flavour profile. Similarly, the literature indicates that functional coatings, whilst maintaining pH, may lead to sensory changes during extended storage [48,49,52].

#### 3.1.3. Water Soluble Dry Matter Values of Strawberries (WSDM)

WSDM (%) indicates the amount of water-soluble dry matter in strawberries and is related to organic acids and other dissolved components, primarily sugars. It is therefore an important quality indicator for determining the fruit’s flavor, consumer appeal and ripeness. Changes in WSDM during storage are directly linked to respiration rate, sugar metabolism, water loss and cellular deterioration [53]. While WSDM values in the control group remained at similar levels on days 0, 7, and 14, they showed a significant decrease, falling to 9.08% on day 21 (*p* < 0.05) (Table S2). This indicates that, in uncoated strawberries, as the post-harvest storage period lengthens, sugars are consumed more rapidly, and the loss of soluble solids increases due to the breakdown of the cellular structure. Similarly, in the GC group, the WSDM value dropped to 8.95% on day 21, and this decrease was found to be statistically significant (*p* < 0.05), suggesting that the protein-based gel coating is not sufficiently protective during long-term storage. In the GCMg group, however, the WSDM values showed a fluctuating trend; the value, which was 9.28% at the start, rose to 11.23% on the 7th day, then fell to 10.08% and 9.32%. This suggests that magnesium initially slowed down metabolism and increased sugar concentration, but its effect diminished over time. Furthermore, water loss, respiratory changes, and the H_2_ gas produced by magnesium’s reaction with water may have played a role in this change by enhancing the solubility of sugars and organic matter [54]. In contrast, in the GCEo group, WSDM values remained stable throughout storage within the range of 10.48–10.83% and showed no significant change (*p* > 0.05). This indicates that the cinnamon essential oil-containing gel coating provides a more stable structure by balancing respiration and sugar metabolism, and also delays spoilage and limits the loss of dissolved solids thanks to its antimicrobial and antioxidant effects [55].

#### 3.1.4. Eh Values of Strawberries

Redox potential (Eh) is an important quality indicator that reflects the oxidative–reductive balance in fruits and provides information on microbial growth and spoilage processes. A high Eh indicates a more oxidative environment, whilst a low Eh indicates a more reducing environment; consequently, it plays a critical role in assessing the oxygen permeability and antioxidant properties of coatings [56]. Upon examination of the results, Eh values in the control group remained unchanged throughout storage from (+)154 to (+)159 mV, indicating that oxidative processes continued unimpeded (*p* > 0.05) (Table S2). In single-component coatings (GC, GCMg, GCEo), Eh values followed a fluctuating trend over time. Although a decrease was observed initially in the GC and GCMg groups, an increase occurred again in the following days, suggesting that the barrier effect of the coating weakened in the long term. The early decrease in the GCMg group may be attributed to H_2_ gas, which is produced by magnesium and exhibits a selective antioxidant effect [24]. In the GCEo group, however, Eh values generally remained lower due to the antioxidant and antimicrobial effects of cinnamon essential oil [57].

The most notable result was obtained in the GCMgEo group; the Eh value generally decreased throughout storage, reaching the lowest level (*p* < 0.05). This indicates that the combined use of magnesium and cinnamon essential oil creates a more reduced and stable environment. This synergistic effect, as noted in the literature, inhibits microbial growth by slowing down oxidative reactions.

#### 3.1.5. Strawberry Weight Loss

Weight loss is a significant quality parameter in strawberries, associated with water loss, respiration, and cellular deterioration, and its increase leads to tissue softening and quality loss [58]. According to the results, weight loss in the control group increased rapidly in the early period and remained at high levels throughout storage (8.45–9%) (Table S2). In the GC group, the loss, which was initially lower, increased as storage progressed and reached its highest value among the coated groups on day 21 (*p* < 0.05). This indicates that the barrier effect of the gel coating weakens over time. In the GCMg group, weight loss was lower and more controlled (3–5.7%), and it is thought that magnesium increases water-holding capacity and limits transpiration. The lowest weight loss was determined in the GCEo group (2.10–4.74%). The semi-permeable barrier created by cinnamon essential oil effectively reduced water vapor transmission. In the GCMgEo group, a moderate but balanced weight loss was observed, indicating that the two components together partially controlled water loss. Indeed, the total weight loss from the beginning (day 7) to the last day of storage was lowest in the GCMgEo group. While the difference in total weight loss observed at the end of storage in the other groups (GCMg and GCEo) was approximately 2.60%, this value remained below 2% in the GCMgEo group.

Overall, weight loss was higher in groups without coating and in those containing only gel, while coatings containing magnesium and essential oils more effectively limited this loss. These findings are consistent with studies reporting that edible coatings reduce weight loss in fruits [39,59,60]. Similarly, chitosan and aloe vera coatings have been reported to reduce weight loss in strawberries [61], while composite films reduce this loss by approximately 5% [62]. Alginate-based multilayer antimicrobial coating reduced weight loss of fresh-cut watermelon during storage [63].

#### 3.1.6. Strawberry Coating Percentage (%)

The coating percentage is an important parameter indicating the adhesion of the edible film to the fruit surface and the continuity of the formed layer. Although a high coating percentage indicates a wider surface coverage, the literature emphasizes that film homogeneity and structural integrity are more decisive in terms of barrier performance [64,65].

In the GC group, the coating percentage was 28.47%; although good adhesion was achieved, quality was not sufficiently maintained during extended storage. In the GCMg group, the highest coating percentage (42.66%) was obtained; however, this was associated with the formation of a thicker and, over time, uneven film (Table S2). In the GCEo (27.26%) and GCMgEo (27.39%) groups, coatings with lower but more homogeneous distribution were obtained, and these groups yielded better results in terms of quality parameters. The results indicate that the coating percentage alone is not sufficient; homogeneous and evenly distributed coatings are more effective. These findings are consistent with studies reporting that coating performance depends on film structure and formulation [43]. Furthermore, it has been noted in the literature that coating efficiency varies depending on the production method and the type of product [66].

### 3.2. Degradation Rate

#### Strawberry Deterioration Rate (%)

**Figure 1:**
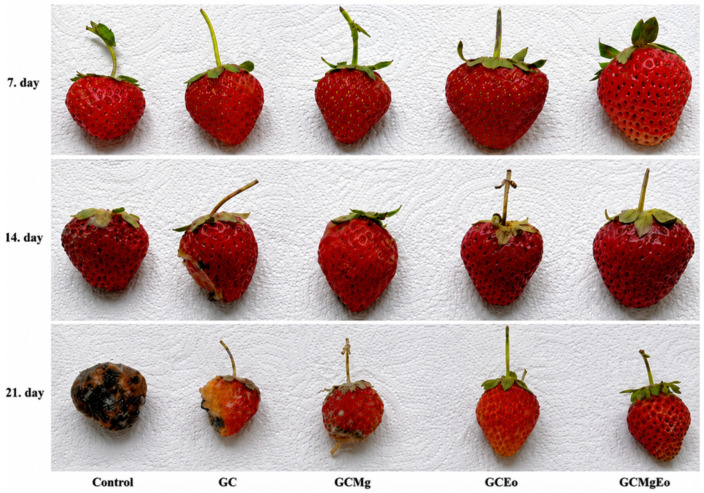
Picture of strawberries deterioration during storage.

Strawberries are fruits with high post-harvest physiological activity and a short shelf life, primarily due to fungal decay [67,68]. The rate of decay is an important indicator for assessing microbial stability. In the control group, spoilage rose to 47.22% by day 14 and to 63.89% by day 21 (*p* < 0.05) (Table S3 and Figure 1). This indicates that microbial growth increases rapidly in uncoated strawberries, which is consistent with the literature [68,69]. In the GC and GCMg groups, spoilage began earlier (on day 7) and reached 100% by day 21. This suggests that plain gel and magnesium-only coatings were unable to sufficiently inhibit microbial growth and that even high coating thickness could create a nutrient medium for microorganisms [70]. In contrast, decay was significantly delayed in the GCEo and GCMgEo groups. The lowest degradation rates were observed in the GCEo group at 19.44% and in the GCMgEo group at 11.11% on day 21 (*p* < 0.05). These results demonstrate that the combination of cinnamon essential oil and magnesium, in particular, provides a strong antimicrobial effect. This effect is explained by essential oils disrupting the cellular structure of microorganisms and inhibiting enzyme activities [39,57]. Studies have shown that coatings containing essential oils extend shelf life and enhance microbial stability by suppressing mould and yeast growth. These findings are consistent with previous reports in the literature [20]. Applying a chitosan coating to freshly cut broccoli demonstrated a bactericidal effect on endogenous *E. coli* and inhibited the growth of total coliform bacteria [71].

### 3.3. HPLC Phenolic Profile Analysis

Table 2 shows the phenolic profile of strawberries. The phenolic profile was evaluated based on two-week data. It was observed that the total phenolic content varied depending on the storage time and the treatment applied. Gallic acid levels in the control group increased from 56.10 µg/g in week 1 to 72.60 µg/g in week 2. Similarly high values were observed in the groups supplemented with magnesium and essential oils. As the storage period increased, the coumaric acid values in essential oil-treated groups (GCEo and GCMgEo) were higher, at 27.73 µg/g and 31.55 µg/g, respectively. Uçurum and Ekşi [72] reported 1.85 mg/kg fresh weight of gallic acid and 0.35–3.76 mg/kg coumaric acid (fresh weight basis) in strawberries. These differences in the composition and quantity of phenolic compounds can be attributed to agronomic practices, cultivar, harvest season, climatic conditions, and fertilization programs. At the beginning of storage, the GCMg group had higher catechin (883.41 µg/g) and epicatechin (183.15 µg/g) values than the other groups. This indicates that magnesium supplementation may promote flavanol accumulation by enhancing phenolic metabolism. Indeed, Engin et al. [40] reported that hydrogen gas was effective in the recovery of phenolic compounds. However, significant decreases in these compounds occurred as storage progressed. This can be explained by the consumption of phenolic substances in oxidative reactions and their susceptibility to natural degradation processes. Catechin concentration varies depending on storage and processing conditions. It is most abundant in green fruits [73]. Catechin is highly sensitive to oxygen. Table 2 shows that the catechin values of the coated groups (GC and GCMg) are particularly high at the beginning of storage. This indicates that the coating gel and magnesium have a significant effect at the beginning of storage. Indeed, Amiri et al. [74] reported that an aloe vera gel-based coating containing nanoencapsulated catechin and calcium chloride preserved the quality of strawberries. Furthermore, Peretto et al. [25] found that essential oils (carvacrol and methyl cinnamate) released from edible films increased the total soluble phenolic content of strawberries.

### 3.4. Texture Analysis

**Figure 2:**
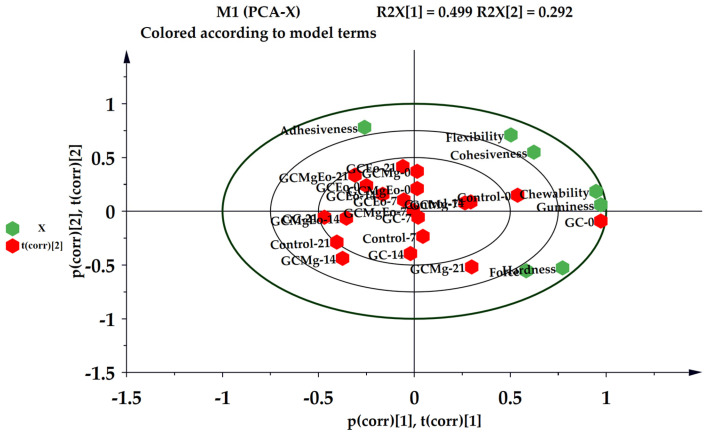
Biplot representation of texture parameters of strawberry groups.

**Figure 3:**
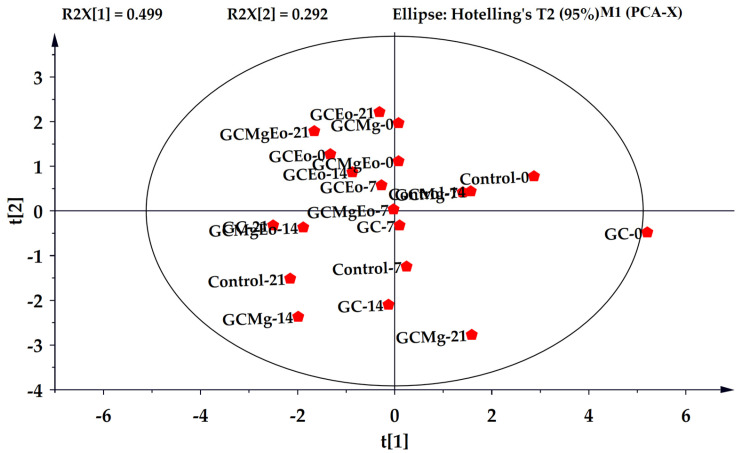
Score scatter plot representation of texture parameters of strawberry groups.

The textural properties of the strawberries are presented in Table S4 and Figure 2 and Figure 3. Among the groups, the magnesium-enriched coating (GCMg) showed significant differences in applied force compared with the control group on days 14 and 21 (*p* < 0.05). At the end of storage, the force value in the GCMg group rose to 1.95 N, whilst a decrease was observed in the other groups. This suggests that magnesium may increase surface hardness. However, this increase is related to increased surface rigidity and adhesiveness rather than tissue preservation. Indeed, higher microbial activity was determined in the GCMg group during the final stages of storage. Hardness is defined as the maximum force required to deform the food to a given extent [75]. Whilst hardness increased in all groups at the start of storage, hardness values decreased in the final period; only in the GCMg group did the increase continue (*p* < 0.05). Although gel-coated (GC) strawberries initially had the highest firmness values, this effect was not maintained until the end of storage. In the groups with added essential oils, the lower firmness was associated with better preservation of tissue integrity. Similarly, it has been reported that coatings based on whey concentrate reduce firmness loss [76] and that chitosan-coated strawberries exhibit firmness similar to that of fresh strawberries [77]. The adhesiveness values ranged from −8 to −37 N·s, with no significant difference observed between the groups (*p* > 0.05). The lowest values were generally observed in the groups to which essential oil had been added, whilst the highest values were seen in the control and GCMg groups. This suggests that cinnamon essential oil preserves tissue integrity, whilst high adhesion values are associated with softening and the formation of a mucous-like structure [78]. Flexibility values were generally close to 1 and showed no significant differences between groups (*p* > 0.05). However, on day 14, the flexibility value of the GCMg group was found to be lower than that of the control group (*p* < 0.05). At the end of storage, the coated groups, particularly those containing essential oil and magnesium, exhibited higher flexibility values compared to the control group. These results indicate that these treatments contribute to the preservation of tissue structure. Similarly, it has been reported that flexibility decreases over time in strawberries stored under modified atmosphere [78]. The groups showed no significant difference in cohesiveness during storage duration (*p* > 0.05). However, the highest values at the end of storage were observed in the groups supplemented with magnesium and essential oils, indicating that the internal structure was better preserved. Similar decreases in cohesiveness during storage have been reported for fruit tissues, where progressive cell wall degradation and loss of cell-to-cell adhesion reduce structural integrity [79]. Chewability and chewiness values differed between groups at the start and end of storage (*p* < 0.05). The groups supplemented with magnesium and essential oil exhibited more stable values, thereby limiting the increase in these parameters. Whilst the highest gumminess and chewability values were initially observed in the GC group, the highest values at the end of storage were found in the GCMg and GCEo groups. Whilst the control group exhibited a more variable profile, the application of magnesium and essential oil more effectively preserved quality by limiting pectin and tissue breakdown. Similar results have been reported for strawberries stored under modified atmosphere [78] and Chinese laurel fruit stored at different temperatures [80].

#### Separating Samples Using PCA

Principal Component Analysis (PCA) was performed to reduce the dimensionality of the dataset containing texture parameters and to identify the principal components (Figure 2 and Figure 3). PCA enabled reduction to a smaller number of components explaining most of the variance in the data. PCA offers the possibility of simplifying the general structure of texture parameters and facilitating their interpretation [41]. In this study, the PCA scores indicate that the strawberries are clearly separated among groups and storage days. The PC1 axis explains 52.2% of the variance, whilst the PC2 axis explains 26.4%. The control group shows higher sensitivity to storage time. Among the edible coating groups, those containing added magnesium and essential oils are positioned closer to the center. This suggests a protective effect of the added components. During storage, the strawberry samples shift towards the left and downwards. Increasing storage duration affects the textural and structural properties of strawberries. From a textural perspective, force, hardness, gumminess and chewability are clustered on the positive (upper) side, whereas flexibility, cohesiveness and stickiness are located on the negative (lower) side.

### 3.5. Sensory Analysis

**Figure 4:**
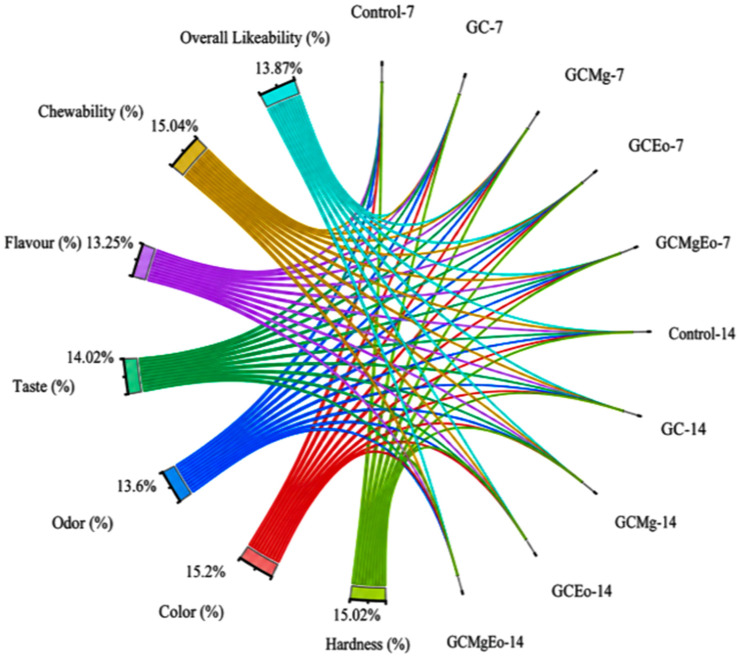
Chord diagram showing the classification of the sensory parameters of strawberries.

Sensory analysis results (weeks 1 and 2) of uncoated (control) and coated strawberries are given in Tables S5 and S6, and the mean percentage values are presented in Table 3. Additionally, Figure 4 shows the chord diagram illustrating the classification of sensory parameters of the strawberries. Due to intense mold growth in the third and fourth weeks, sensory analyses were limited to the first two weeks. In the first two weeks, the control group generally showed higher sensory scores than the coated groups. This may be related to the coatings delaying ripening and slowing down the senescence process. Firmness, color, odor, taste, aroma, chewability, and overall liking values varied throughout the 14-day storage period. While the control group showed more variation in hardness values, coatings containing Mg and cinnamon essential oil yielded more stable results [77,81,82]. Color changes generally increased with ripening, but were statistically insignificant in most groups [83,84].

Coating type had an effect on odor, taste, and aroma scores, with Mg and Eo additions partially suppressing some sensory characteristics. This effect can be explained by the influence of essential oils on fruit odor and taste [84,85,86]. Chewability values were found to be consistent with hardness, with the control group showing higher values and the groups containing Mg and Eo showing lower values [80]. In terms of overall liking scores, the control group had the highest values, while the GCMgEo group had the lowest. The GC group gave the closest results to the control group. Coating applications generally maintained sensory quality during storage, but the addition of Mg and essential oils had a negative effect on some sensory parameters [77,84].

Overall, the incorporation of magnesium (Mg) and essential oils into the coating affected the sensory and textural properties of the strawberries. Due to their high volatility, essential oil compounds are mainly released into the headspace surrounding the fruit during storage. This helps suppress undesirable odors and limits microbial growth. Kavrut and Sezer [17] reported that edible films containing thyme essential oil showed antimicrobial activity in meatballs. Similarly, Quesada et al. [87] demonstrated that active packaging containing thyme essential oil improved the microbial quality of cooked pork slices, while Tančinová et al. [88] reported that essential oils in the vapor phase effectively reduced fungal growth on strawberries.

In addition, hydrogen (H_2_) produced when Mg reacts with moisture on the fruit surface has been reported to possess antioxidant and reducing properties, which may help preserve the texture and sensory quality of foods during storage. Kavrut et al. [89] found that edible coatings containing whey protein, thyme extract, and magnesium reduced lipid oxidation and proteolysis, leading to improvements in the volatile compound profile as well as the nutritional and microbiological quality of the product. Although these findings were obtained from meat products, similar mechanisms may also have contributed to the quality preservation observed in the coated strawberries in the present study.

According to this classification, the sensory parameters of strawberries over a two-week period (firmness, color, odor, taste, aroma, texture and overall acceptability) showed variable trends. The color parameter accounted for the highest proportion (15.20%), followed by texture (15.04%) and firmness (15.02%). The lowest value was observed for aroma (13.25%).

### 3.6. A Comparison of Edible Coating Performance on Different Fruits

Edible coatings have been extensively studied to preserve the postharvest quality of various fruits using different natural biopolymers. In apples, alginate and gum arabic coatings effectively reduced weight loss, delayed browning, maintained firmness, and extended storage life [90,91]. Similarly, chitosan-based coatings applied to apricots and mangoes improved fruit quality by reducing moisture loss, slowing ripening, preserving physicochemical properties, and extending shelf life [92,93]. These findings show that coating materials can be selected based on the physiological characteristics of different fruit types. Similarly, strawberries coated with chitosan or aloe vera have reduced microbial growth and weight loss while maintaining better color, firmness, and overall quality during storage [49,94]. Alginate-based coatings have also shown promising results in peaches and pineapples by reducing respiration rates, protecting the tissue, and delaying quality deterioration throughout storage [95,96]. Overall, these studies demonstrate that edible coatings provide an effective strategy for preserving postharvest quality, reducing spoilage, and extending the shelf life of a wide variety of fresh fruits [97,98].

## 4. Conclusions

In this study, the effect of adding magnesium and cinnamon essential oil to whey protein isolate (WPI)-based edible coatings on the quality of strawberries during storage was investigated. The results clearly demonstrated that the coatings delayed post-harvest deterioration in the strawberries. Whilst weight loss, color fading, softening and microbial spoilage occurred more rapidly in strawberries without coatings, these changes were significantly slowed in the coated groups. In particular, coatings containing both magnesium and cinnamon essential oil demonstrated the best performance. Color, physicochemical and textural analyses revealed that this combination better preserved the strawberries’ brightness, stability and structural properties. Furthermore, the coatings helped to preserve moisture by reducing water loss. The fact that the redox potential remained at lower levels in the magnesium-containing coatings suggests the creation of a more reducing environment. Cinnamon essential oil, meanwhile, delayed spoilage thanks to its strong antimicrobial effect.

From a sensory perspective, the coatings-controlled ripening more effectively; however, a decrease in taste and aroma intensity was observed in samples containing magnesium and cinnamon oil. Overall, the coatings preserved strawberry quality. In conclusion, WPI-based coatings enriched with magnesium and cinnamon essential oil emerge as an effective and environmentally friendly alternative for extending the shelf life of strawberries.

## Figures and Tables

**Table 1:** Color analysis values of strawberry groups.

		Storage Time (Days)			
Parameters	Applications	0	7	14	21
L*	Control	33.44 ± 2.06 ^aA^	31.04 ± 1.31 ^abB^	30.77 ± 1.47 ^aB^	30.10 ± 1.31 ^aB^
	GC	29.25 ± 1.96 ^bA^	29.34 ± 2.21 ^bA^	30.14 ± 2.02 ^aA^	25.44 ± 0.63 ^bB^
	GCMg	29.89 ± 1.61 ^bAB^	30.97 ± 2.11 ^abAB^	31.83 ± 1.35 ^aA^	29.17 ± 1.13 ^aB^
	GCEo	31.57 ± 2.32 ^abAB^	31.97 ± 1.22 ^aA^	32.33 ± 2.16 ^aA^	29.42 ± 2.13 ^aB^
	GCMgEo	31.66 ± 2.80 ^abA^	31.47 ± 2.60 ^abA^	31.51 ± 1.47 ^aA^	30.22 ± 0.35 ^aA^
a*	Control	21.16 ± 2.68 ^aA^	20.51 ± 4.70 ^aA^	19.99 ± 4.63 ^aA^	19.83 ± 3.56 ^aA^
	GC	23.45 ± 2.86 ^aA^	22.40 ± 3.55 ^aAB^	21.25 ± 1.04 ^aAB^	19.86 ± 0.99 ^aB^
	GCMg	21.31 ± 1.24 ^aA^	20.83 ± 4.13 ^aA^	20.52 ± 3.15 ^aA^	20.32 ± 0.65 ^aA^
	GCEo	22.62 ± 3.53 ^aA^	21.12 ± 2.02 ^aA^	21.08 ± 3.42 ^aA^	20.92 ± 1.74 ^aA^
	GCMgEo	22.16 ± 6.17 ^aA^	22.05 ± 2.65 ^aA^	21.05 ± 1.08 ^aA^	20.59 ± 0.73 ^aA^
b*	Control	12.65 ± 4.94 ^aA^	11.46 ± 5.50 ^aA^	11.04 ± 4.41 ^aA^	10.94 ± 2.37 ^aA^
	GC	11.99 ± 3.05 ^aA^	11.36 ± 3.88 ^aA^	9.91 ± 2.12 ^aA^	8.93 ± 0.57 ^abA^
	GCMg	10.70 ± 1.13 ^aA^	10.68 ± 2.52 ^aA^	9.90 ± 1.89 ^aA^	8.79 ± 0.95 ^abA^
	GCEo	11.37 ± 4.15 ^aA^	12.10 ± 4.37 ^aA^	11.10 ± 5.13 ^aA^	7.87 ± 3.64 ^abA^
	GCMgEo	12.38 ± 4.21 ^aA^	11.45 ± 4.22 ^aA^	11.15 ± 4.48 ^aA^	7.42 ± 3.48 ^bA^
C*	Control	23.95 ± 5.44 ^aA^	23.12 ± 7.30 ^aA^	24.29 ± 7.09 ^aA^	23.41 ± 7.70 ^aA^
	GC	26.40 ± 3.53 ^aA^	24.11 ± 6.35 ^aAB^	19.22 ± 5.10 ^aB^	19.06 ± 1.07 ^aB^
	GCMg	16.74 ± 5.69 ^bB^	24.93 ± 6.26 ^aA^	22.63 ± 5.22 ^aAB^	22.66 ± 2.58 ^aAB^
	GCEo	23.08 ± 5.93 ^bA^	23.06 ± 5.10 ^aA^	21.30 ± 7.72 ^aA^	22.46 ± 2.72 ^aA^
	GCMgEo	25.31 ± 7.43 ^aA^	23.16 ± 6.39 ^aA^	22.43 ± 5.72 ^aA^	22.58 ± 0.97 ^aA^
h°	Control	31.03 ± 7.36 ^aA^	28.41 ± 6.30 ^aA^	28.07 ± 5.49 ^abA^	29.94 ± 2.12 ^aA^
	GC	26.80 ± 5.26 ^aA^	27.52 ± 3.95 ^aA^	25.80 ± 4.74 ^bA^	25.22 ± 6.42 ^bA^
	GCMg	30.11 ± 0.79 ^aA^	31.81 ± 6.56 ^aA^	32.53 ± 4.85 ^aA^	34.24 ± 3.06 ^aA^
	GCEo	29.14 ± 5.94 ^aA^	31.40 ± 5.70 ^aA^	31.62 ± 5.41 ^abA^	31.72 ± 0.72 ^aA^
	GCMgEo	29.09 ± 3.20 ^aA^	29.26 ± 4.87 ^aA^	29.35 ± 5.37 ^abA^	31.46 ± 2.47 ^aA^
∆E	Control	0.00 ± 0.00 ^B^	9.13 ± 6.00 ^aA^	9.28 ± 6.02 ^abA^	8.31 ± 2.61 ^aA^
	GC	0.00 ± 0.00 ^C^	5.35 ± 1.83 ^aB^	6.06 ± 4.11 ^bAB^	8.96 ± 1.93 ^aA^
	GCMg	0.00 ± 0.00 ^B^	8.07 ± 4.63 ^aA^	9.30 ± 4.74 ^abA^	9.60 ± 2.56 ^aA^
	GCEo	0.00 ± 0.00 ^B^	8.44 ± 3.71 ^aA^	11.02 ± 1.23 ^abA^	8.98 ± 6.19 ^aA^
	GCMgEo	0.00 ± 0.00 ^B^	8.97 ± 2.54 ^aA^	12.70 ± 3.49 ^aA^	12.99 ± 7.36 ^aA^

^a,b^: Means denoted by the same lowercase letter in the same column are statistically indistinguishable (by treatment). ^A,B^: Means denoted by the same uppercase letter in the same row are statistically indistinguishable (by storage duration). Significant (*p* < 0.05); not significant (*p* > 0.05).

**Table 2:** Effect of different edible coatings on the phenolic profile in strawberry.

Applications	Storage Time (Weeks)	Phenolic Profile (µg/g Dry Extract)			
		Gallic Acid	*p*-Coumaric Acid	Catechin	Epicatechin
Control	1	56.10	51.47	481.71	148.27
	2	72.60	35.47	554.40	117.91
GC	1	45.60	60.24	818.36	155.81
	2	31.42	42.76	422.36	109.47
GCMg	1	23.85	55.38	883.41	183.15
	2	43.91	26.10	236.26	56.60
GCEo	1	34.29	23.20	218.56	47.61
	2	35.76	27.73	199.34	nd
GCMgEo	1	25.13	27.31	217.42	43.72
	2	29.24	31.55	314.00	nd

GC: gel coating, GCMg: gel coating with added magnesium, GCEo: gel coating with added cinnamon essential oil, GCMgEo: gel coating with added magnesium and essential oil. nd: Not detected.

**Table 3:** Mean percentage values for the sensory analysis parameters of strawberries.

Applications	Hardness (%)	Color (%)	Odor (%)	Taste (%)	Flavour (%)	Chewability (%)	Overall Likeability (%)
Control-7	13.23	13.09	14.32	15.42	14.32	15.02	14.59
GC-7	14.72	15.42	13.58	13.28	14.00	14.58	14.42
GCMg-7	15.24	13.95	13.75	13.95	13.39	15.60	14.13
GCEo-7	14.78	15.27	13.41	14.26	12.92	15.43	13.93
GCMgEo-7	15.38	16.38	13.51	13.74	12.71	15.38	12.91
Control-14	14.92	13.98	13.43	13.98	13.98	14.79	14.92
GC-14	14.76	14.93	13.44	14.60	12.93	14.93	14.42
GCMg-14	15.82	15.32	13.30	14.14	12.80	15.16	13.46
GCEo-14	15.81	15.48	13.60	13.60	12.76	15.48	13.27
GCMgEo-14	15.51	18.17	13.68	13.25	12.65	14.08	12.65
Mean (%)	15.02	15.20	13.60	14.02	13.25	15.04	13.87

## Data Availability

The original contributions presented in this study are included in the article/Supplementary Materials. Further inquiries can be directed to the corresponding author.

## Supplementary Materials

The following supporting information can be downloaded at: https://www.mdpi.com/article/10.3390/foods15142534/s1, Table S1: Sensory Analysis Evaluation Form of Strawberry; Table S2: Results of Physicochemical Analyses of Strawberry Groups; Table S3: Results of the Deterioration Rate (%) for Strawberry Groups; Table S4: Texture Analysis Results of Strawberry Groups; Table S5: Sensory Analysis Results for the First Week of Strawberries; Table S6: Sensory Analysis Results for the Second Week of Strawberries.
