# Supplementary material for: Effects of Magnesium- and Cinnamon Essential Oil-Enriched Edible Gel Coatings on the Quality Parameters of Strawberries
Source: Foods. 2026 Jul 17;15(14):2534. doi: 10.3390/foods15142534 (PMC13408616; doi:10.3390/foods15142534)
Supplement: Supplementary file 1 [file foods-15-02534-s001.zip › foods-4402265-supplementary.pdf]

Table S1. Sensory analysis evaluation form of strawberry.

No	Hardness	Color	Odor	Taste	Flavour	Chewability	Overall Appeal
1							
2							
3							
4							
5							
6							
7							
8							
9							

In this forum, you will be asked to share your thoughts on the sensory characteristics of strawberries samples.
Dear Participants;

- Before starting, please rinse your mouth with the water provided.
- After tasting each sample, remember to rinse your mouth with water.
- You do not have to swallow the food samples. You may use the spittoons and napkins provided.
- You will evaluate the strawberries samples, which have been assigned different codes, in terms of the following characteristics: color, smell, taste, aroma, chewiness, and overall acceptability. This evaluation is done by assigning a number from 1 to 9. Simply mark the appropriate score with an X.
- **Gel Coating Taste, Gel Coating Odor: Perceptible, Not Perceptible**
- 1: Extremely poor;
- 2: Very poor;
- 3: Poor;
- 4: Below average;
- 5: Average;
- 6: Above average;
- 7: Good;
- 8: Very good;
- 9: Excellent

Table S2. Results of physicochemical analyses of strawberry groups.

		Storage Time (days)			
**Parameters**	Applications	0	7	14	21
**pH**	Control	3.49±0.17^{bcAB}	3.53±0.17^{bcA}	3.32±0.12^{cB}	3.49±0.07^{cAB}
	GC	3.43±0.10^{cB}	3.47±0.05^{cB}	3.44±0.09^{bB}	3.64±0.07^{bcA}
	GCMg	3.65±0.14^{aA}	3.67±0.05^{aA}	3.61±0.13^{aA}	3.67±0.25^{bA}
	GCEo	3.58±0.04^{abcA}	3.60±0.10^{abA}	3.57±0.09^{aA}	3.58±0.05^{bcA}
	GCMgEo	3.61±0.14^{abB}	3.63±0.06^{abB}	3.61±0.05^{aB}	3.99±0.09^{aA}
**Eh (mV)**	Control	+159.17±4.92^{cA}	+154.67±3.39^{aA}	+156.83±2.14^{bA}	+159.50±6.83^{bA}
	GC	+165.83±7.44^{bcA}	+145.33±24.77^{aB}	+168.33±9.75^{aA}	+169.00±6.51^{aA}
	GCMg	+175.67±11.08^{aA}	+141.83±9.28^{aB}	+169.00±6.07^{aA}	+172.67±2.25^{aA}
	GCEo	+167.33±4.41^{abcA}	+149.50±9.12^{aB}	+161.50±6.66^{abA}	+145.17±3.57^{cB}
	GCMgEo	+169.33±4.37^{abA}	+148.83±3.19^{aB}	+156.83±12.27^{bB}	+136.67±2.94^{dC}
**WSDM (%)**	Control	10.77±0.89^{aA}	10.94±1.09^{aA}	11.22±1.11^{abA}	9.08±0.89^{bB}
	GC	10.51±1.23^{aA}	10.42±0.74^{aA}	10.15±0.39^{bA}	8.95±0.68^{bB}
	GCMg	9.28±0.45^{aB}	11.23±0.79^{aA}	10.08±1.22^{bB}	9.32±0.86^{bB}
	GCEo	10.58±1.80^{aA}	10.48±0.74^{aA}	10.83±0.83^{abA}	10.58±1.05^{aA}
	GCMgEo	11.28±1.52^{aA}	11.13±0.51^{aA}	11.33±0.87^{aA}	11.12±0.35^{aA}
**Weight Loss (%)**	Control	0.00±0.00	8.45±1.48^{aA}	8.51±2.11^{aA}	9.00±1.43^{aA}
	GC	0.00±0.00	4.81±0.65^{bC}	5.83±0.49^{bB}	8.80±0.99^{aA}
	GCMg	0.00±0.00	3.11±0.62^{cdB}	3.17±0.27^{cB}	5.72±1.84^{bA}
	GCEo	0.00±0.00	2.10±1.10^{dB}	2.86±0.86^{cB}	4.74±0.87^{bA}
	GCMgEo	0.00±0.00	3.98±0.90^{bcB}	4.98±1.54^{bAB}	5.91±1.38^{bA}
**Coating Percentage (%)**	Control	0.00±0.00			
	GC	28.47±11.27			
	GCMg	42.66±5.85			
	GCEo	27.26±4.62			
	GCMgEo	24.39±2.68			

$^{a–d}$: Means denoted by the same lowercase letter in the same column are statistically indistinguishable from one another (by treatment). $^{A–C}$: Means denoted by the same uppercase letter in the same row are statistically indistinguishable from one another (by storage duration). * significant ($p < 0.05$), not significant ($p > 0.05$).

Table S3. Results of the deterioration rate (%) for strawberry groups.

		Storage Time (days)			
**Parameters**	Applications	0	7	14	21
**Deterioration Rate (%)**	Control	0.00±0.00^{C}	0.00±0.00^{cB}	47.22±12.55^{bB}	63.89±16.39^{bA}
	GC	0.00±0.00^{C}	5.56±13.61^{abC}	66.67±27.89^{aB}	100.00±0.00^{aA}
	GCMg	0.00±0.00^{D}	13.89±12.55^{aC}	55.56±13.61^{abB}	100.00±0.00^{aA}
	GCEo	0.00±0.00^{B}	0.00±0.00^{bB}	0.00±0.00^{cB}	19.44±6.80^{cA}
	GCMgEo	0.00±0.00^{B}	0.00±0.00^{bB}	0.00±0.00^{cB}	11.11±8.61^{cA}

$^{a–c}$: Means denoted by the same lowercase letter in the same column are statistically indistinguishable from one another (by treatment). $^{A–D}$: Means denoted by the same uppercase letter in the same row are statistically indistinguishable from one another (by storage duration). * significant ($p < 0.05$), not significant ($p > 0.05$).

Table S4. Texture analysis results of strawberry groups.

		Storage Time (days)			
**Parameters**	Applications	0	7	14	21
**Force (N)**	Control	1.21±0.65^{aA}	1.45±0.68^{aA}	1.36±0.54^{aA}	1.12±0.22^{bA}
	GC	1.53±0.78^{aA}	1.34±0.22^{aA}	1.17±0.47^{abA}	1.07±0.11^{bA}
	GCMg	0.94±0.40^{aB}	1.03±0.27^{aB}	0.83±0.36^{bB}	1.95±0.84^{aA}
	GCEo	0.93±0.45^{aA}	1.16±0.61^{aA}	1.06±0.25^{abA}	0.88±0.47^{bA}
	GCMgEo	0.97±0.62^{aA}	1.17±0.31^{aA}	1.09±0.31^{abA}	0.84±0.14^{bA}
**Hardness (N)**	Control	200±0.64^{bA}	2.20±0.79^{aA}	1.90±0.89^{abA}	1.60±0.42^{bA}
	GC	2.88±0.74^{aA}	2.20±0.94^{aAB}	2.09±0.75^{aAB}	1.36±0.51^{bB}
	GCMg	1.34±0.45^{bB}	2.00±0.49^{aAB}	1.53±0.53^{abB}	2.49±0.82^{aA}
	GCEo	1.27±0.49^{bA}	1.53±0.27^{aA}	1.21±0.41^{bA}	1.09±0.43^{bA}
	GCMgEo	1.87±0.54^{bA}	1.95±0.45^{aA}	1.16±0.20^{bB}	0.93±0.23^{bB}
**Adhesiveness (N·s)**	Control	-23.13±22.72^{aA}	-16.66±18.40^{aA}	-14.99±6.85^{aA}	-20.86±23.98^{aA}
	GC	-26.46±20.99^{aA}	-8.76±4.12^{aA}	-33.31±33.60^{aA}	-11.45±15.99^{aA}
	GCMg	-10.98±3.61^{aA}	-21.61±9.48^{aAB}	-36.71±13.38^{aB}	-30.34±22.95^{aB}
	GCEo	-8.99±5.32^{aA}	-12.92±5.77^{aA}	-15.06±20.97^{aA}	-8.84±7.41^{aA}
	GCMgEo	-8.07±1.57^{aA}	-13.05±6.85^{aA}	-17.87±18.92^{aA}	-9.86±13.40^{aA}
**Flexibility**	Control	1.16±0.83^{aA}	0.82±0.32^{aA}	0.96±0.02^{aA}	0.66±0.44^{aA}
	GC	1.01±0.55^{aA}	0.95±0.04^{aA}	0.53±0.46^{abA}	0.83±0.32^{aA}
	GCMg	1.15±0.45^{aA}	0.98±0.03^{aA}	0.30±0.35^{bA}	0.91±1.29^{aA}
	GCEo	0.98±0.02^{aA}	0.97±0.03^{aA}	1.00±0.22^{aA}	0.97±0.04^{aA}
	GCMgEo	1.05±0.15^{aA}	0.97±0.03^{aAB}	0.70±0.44^{abB}	0.98±0.03^{aAB}
**Cohesiveness**	Control	0.21±0.14^{aA}	0.12±0.07^{aA}	0.21±0.12^{aA}	0.08±0.03^{aA}
	GC	0.24±0.16^{aA}	0.11±0.02^{aB}	0.11±0.08^{aB}	0.08±0.00^{aB}
	GCMg	0.19±0.15^{aA}	0.20±0.18^{aA}	0.15±0.25^{aA}	0.12±0.10^{aA}
	GCEo	0.15±0.05^{aA}	0.15±0.05^{aA}	0.16±0.71^{aA}	0.23±0.21^{aA}
	GCMgEo	0.15±0.03^{aA}	0.13±0.03^{aA}	0.13±0.10^{aA}	0.18±0.19^{aA}
**Guminess (N)**	Control	0.39±0.26^{abA}	0.27±0.19^{aA}	0.36±0.21^{aA}	0.13±0.06^{aA}
	GC	0.59±0.21^{aA}	0.25±0.11^{aB}	0.26±0.12^{aB}	0.11±0.04^{bB}
	GCMg	0.27±0.27^{bA}	0.37±0.16^{aA}	0.17±0.25^{aA}	0.27±0.20^{aA}
	GCEo	0.18±0.08^{bA}	0.24±0.11^{aA}	0.21±0.14^{aA}	0.26±0.26^{aA}
	GCMgEo	0.27±0.07^{bA}	0.25±0.08^{aA}	0.16±0.15^{aA}	0.17±0.16^{aA}
**Chewability (N.mm)**	Control	0.61±0.84^{aA}	0.21±0.19^{aA}	0.34±0.20^{aA}	0.10± 0.09^{aA}
	GC	0.64±0.49^{aA}	0.24±0.10^{aB}	0.20±0.16^{aB}	0.09±0.06^{aB}
	GCMg	0.29±0.24^{aA}	0.36±0.15^{aA}	0.12±0.27^{aA}	0.27±0.49^{aA}
	GCEo	0.18±0.07^{aA}	0.24±0.11^{aA}	0.19±0.11^{aA}	0.26±0.26^{aA}
	GCMgEo	0.29±0.07^{aA}	0.25±0.08^{aA}	0.13±0.15^{aA}	0.16±0.15^{aA}

^{a,b}: Means denoted by the same lowercase letter in the same column are statistically indistinguishable from one another (by treatment). ^{A,B}: Means denoted by the same uppercase letter in the same row are statistically indistinguishable from one another (by storage duration). * significant ($p < 0.05$), not significant ($p > 0.05$).

Table S5. Sensory analysis results for the first week of strawberries.

Applications	Hardness	Color	Odor	Taste	Flavour	Chewability	Overall Likeability
Control	$6.86{\pm}2.35^{ab}$	$6.79{\pm}2.52^{ab}$	$7.43{\pm}1.34^{a}$	$8.00{\pm}0.88^{a}$	$7.43{\pm}1.50^{a}$	$7.79{\pm}1.37^{a}$	$7.57{\pm}1.34^{a}$
GC	$7.36{\pm}0.84^{a}$	$7.71{\pm}1.14^{a}$	$6.79{\pm}1.19^{ab}$	$6.64{\pm}1.00^{ab}$	$7.00{\pm}1.30^{a}$	$7.29{\pm}1.14^{ab}$	$7.21{\pm}1.12^{ab}$
GCMg	$5.93{\pm}1.49^{ab}$	$5.43{\pm}1.99^{b}$	$5.35{\pm}2.06^{bc}$	$5.43{\pm}2.10^{bc}$	$5.21{\pm}1.76^{b}$	$6.07{\pm}1.64^{bc}$	$5.50{\pm}1.79^{c}$
GCEo	$6.29{\pm}2.33^{ab}$	$6.50{\pm}1.61^{ab}$	$5.71{\pm}2.13^{bc}$	$6.07{\pm}1.94^{bc}$	$5.50{\pm}2.31^{b}$	$6.57{\pm}1.95^{abc}$	$5.93{\pm}2.56^{bc}$
GCMgEo	$5.36{\pm}2.27^{b}$	$5.71{\pm}2.27^{b}$	$4.71{\pm}2.16^{c}$	$4.79{\pm}2.67^{c}$	$4.43{\pm}2.24^{b}$	$5.36{\pm}1.86^{c}$	$4.50{\pm}2.53^{c}$

GC: gel coating, GCMg: gel coating with added magnesium, GCEo: gel coating with added cinnamon essential oil, GCMgEo: gel coating with added magnesium and essential oil. $^{a–c}$: Means denoted by the same lowercase letter in the same column are statistically indistinguishable from one another (by treatment). * significant ($p < 0.05$), not significant ($p > 0.05$).

Table S6. Sensory analysis results for the second week of strawberries.

Applications	Hardness	Color	Odor	Taste	Flavour	Chewability	Overall Likeability
Control	$7.93{\pm}0.62^{a}$	$7.43{\pm}1.55^{a}$	$7.14{\pm}1.35^{a}$	$7.43{\pm}1.87^{a}$	$7.43{\pm}1.22^{a}$	$7.86{\pm}1.03^{a}$	$7.93{\pm}0.83^{a}$
GC	$6.36{\pm}1.74^{b}$	$6.43{\pm}1.83^{a}$	$5.79{\pm}2.01^{ab}$	$6.29{\pm}1.44^{ab}$	$5.57{\pm}1.79^{b}$	$6.43{\pm}1.16^{a}$	$6.21{\pm}1.63^{b}$
GCMg	$6.71{\pm}1.59^{ab}$	$6.50{\pm}1.95^{a}$	$5.64{\pm}2.37^{ab}$	$6.00{\pm}1.84^{abc}$	$5.43{\pm}2.31^{b}$	$6.43{\pm}2.03^{a}$	$5.71{\pm}2.23^{bc}$
GCEo	$6.64{\pm}2.41^{ab}$	$6.50{\pm}1.79^{a}$	$5,71{\pm}2.02^{ab}$	$5.71{\pm}2.30^{bc}$	$5.36{\pm}2.53^{b}$	$6.50{\pm}2.10^{a}$	$5.57{\pm}2.59^{bc}$
GCMgEo	$5.43{\pm}2.44^{b}$	$6.36{\pm}1.55^{a}$	$4.79{\pm}2.36^{b}$	$4.64{\pm}2.34^{c}$	$4.43{\pm}2.21^{b}$	$4.93{\pm}2.36^{b}$	$4.43{\pm}2.17^{c}$

GC: gel coating, GCMg: gel coating with added magnesium, GCEo: gel coating with added cinnamon essential oil, GCMgEo: gel coating with added magnesium and essential oil. $^{a–c}$: Means denoted by the same lowercase letter in the same column are statistically indistinguishable from one another (by treatment). * significant ($p < 0.05$), not significant ($p > 0.05$).
